# What supervisors say in their feedback: construction of CanMEDS roles in workplace settings

**DOI:** 10.1007/s10459-015-9634-9

**Published:** 2015-09-05

**Authors:** Nienke Renting, Tim Dornan, Rijk O. B. Gans, Jan C. C. Borleffs, Janke Cohen-Schotanus, A. Debbie C. Jaarsma

**Affiliations:** Center for Educational Development and Research in Health Professions, University Medical Center Groningen and University of Groningen, Groningen, The Netherlands; Department of Internal Medicine, University Medical Center Groningen and University of Groningen, Groningen, The Netherlands; Centre for Medical Education, Queen’s University Belfast, Belfast, UK; Department of Education Development and Research, Maastricht University, Maastricht, The Netherlands

**Keywords:** CanMEDS, Competency-based medical education, Feedback, Discourse analysis, Patient-centred care, Postgraduate training, Residency, Workplace learning

## Abstract

The CanMEDS framework has been widely adopted in residency education and feedback processes are guided by it. It is, however, only one of many influences on what is actually discussed in feedback. The sociohistorical culture of medicine and individual supervisors’ contexts, experiences and beliefs are also influential. Our aim was to find how CanMEDS roles are constructed in feedback in a postgraduate curriculum-in-action. We applied a set of discourse analytic tools to written feedback from 591 feedback forms from 7 hospitals, including 3150 feedback comments in which 126 supervisors provided feedback to 120 residents after observing their performance in authentic settings. The role of Collaborator was constructed in two different ways: a cooperative discourse of equality with other workers and patients; and a discourse, which gave residents positions of power—delegating, asserting and ‘taking a firm stance’. Efficiency—being fast and to the point emerged as an important attribute of physicians. Patients were seldom part of the discourses and, when they were, they were constructed as objects of communication and collaboration rather than partners. Although some of the discourses are in line with what might be expected, others were in striking contrast to the spirit of CanMEDS. This study’s findings suggest that it takes more than a competency framework, evaluation instruments, and supervisor training to change the culture of workplaces. The impact on residents of training in such demanding, efficiency-focused clinical environments is an important topic for future research.

## Introduction

In order to prepare physicians better for their future practice and, ultimately, increase the quality of care, competency frameworks have been widely adopted. The assumption that defining professional standards for educational purposes can lead to better care is made explicit in the slogan of the CanMEDS framework: ‘Better standards, better physicians, better care’ (Frank 2005). Competencies concerning team processes like communication, collaboration and management, have been included alongside medical expertise in response to concerns about patient safety and the increasing complexity of healthcare systems. The rationale for including these competencies within competency frameworks is supported by the findings of multiple review articles that improved performance in those domains correlated with a higher quality of care (Dietz et al. 2014; Kuenzle et al. 2010; Schmutz and Manser 2013). Showing a correlation between physician attributes and quality of care, however, does not guarantee that including those attributes in competency frameworks will improve care.

Although CanMEDS is the most widely adopted competency framework, little research has been conducted on how curricula informed by it operate in practice. Previous research has focused mainly on the development of assessment instruments (Hall et al. 2012; Jefferies et al 2011; Sherbino et al. 2013), implementation processes (Frank et al. 2010; Iglar et al. 2013; Ringsted et al. 2006; Rousseau et al. 2007; Scheele et al. 2008) and the extent to which graduates feel prepared for practice (Berkenbosch et al. 2013; Card et al. 2006; Haji and Steven 2014). Given that competency-based education directs attention to learning outcomes, it has seemed logical that research should concentrate on developing valid and reliable assessment instruments to measure these outcomes. Formative evaluation of performance in practice, which can show whether trainees are able to manage the complexities of practice, is also a powerful learning tool given that it provides learners with feedback about their performance (van der Vleuten et al. 2010; Ross et al. 2012). Since residents’ knowledge and practice is influenced by the feedback they receive, the way supervisors operationalize the CanMEDS roles in their feedback is an important influence on patient care. Despite its strong influence on what residents consider to be high quality care and their trajectories towards becoming competent physicians, we have not been able to find any research into what supervisors actually say in their feedback on CanMEDS roles.

Even though the roles and their underlying competencies have been described in great detail in official documents, that does not mean they have the same meaning in practice. Supervisors’ conceptions of the roles are not only based on their knowledge of official documents, but also on their own experiences and beliefs, which are in potential conflict with the official CanMEDS discourse. Whitehead et al. (2011) performed a critical discourse analysis on the historical development of these official documents, concluding that the roles should be treated as socially negotiated outcomes rather than objective ideals; the roles, in other words, fit the needs of the profession and society at a certain time and in specific contexts. This insight applies at the level of single training programmes as well as the (inter)national level. Their meaning may change as the roles are translated into practice so that they support a different kind of health care from that proposed in the original framework. Zibrowski et al. (2009) found that residents had a narrow view of the CanMEDS roles and sometimes did not see their relevance to patient care so it is relevant to study how supervisors construct the roles in their feedback to residents and how this relates to the original aims of the framework. We set out to explore how CanMEDS-based postgraduate training runs through in practice, by analysing how the roles are constructed in a curriculum-in-action. By studying how supervisors constructed the CanMEDS roles in their written feedback to residents, we aimed to gain insight into what is considered ‘natural’ in an authentic healthcare setting.

## Conceptual orientation

### Constructivist stance

Setting out to analyse how the CanMEDS roles are constructed in workplace education places this research firmly within the constructivist theoretical paradigm. A constructivist stance legitimates the actuality of the CanMEDS roles as constructed in social interactions, specifically supervisors’ feedback to residents. This constructivist stance departs from previous research (Bordage 2009), which has tended not to specify its theoretical orientation (Beckman and Cook 2007), but tacitly adopted behaviourist (assessment drives learning) or cognitivist (how learners deal with feedback) assumptions. A behaviourist stance would make it logical to improve practice and performance by developing assessment instruments, whereas a cognitivist stance might focus on intra-individual responses to feedback. We reasoned that a constructivist stance would complement those other approaches by focusing on how meaning is constructed during feedback-in-action.

### A critical discourse approach

Our constructivist stance towards the construction of professional roles makes it logical to choose one of the many discourse methodologies. A critical discourse approach assumes that the language people use is influenced by their personal social backgrounds, cultures, and beliefs. People’s spoken words construct social positions and actions by making one stance or action seem natural and an alternative one less natural. In the context of our study, this means that what supervisors say in their feedback is based not just upon what they have read about the CanMEDS roles and their training, but also upon their personal histories and experiences. The word ‘Critical’, as applied to discourse analysis, means that the analysis explores how language can reveal relationships, power, and meaning that may otherwise remain hidden (Fairclough 2001). It explores how language relates to the social construction of various phenomena; how it functions in maintaining and changing relations and ideologies (Fairclough 2001). Critical discourse analysis is used widely across many disciplines, and is increasingly acknowledged as an insightful methodology for medical education research (Hodges et al. 2014; Dornan 2014). Critical discourse approaches informed by the work of Foucault (MacLeod 2011), Bakhtin (Dornan et al. 2015) and Gee (Graham and Dornan 2013) have all been used in medical education.

### The present study

The objective of the present study was to find out what is considered important in a clinical setting in which the curriculum is informed by the CanMEDS framework. We chose to use a methodology developed by Gee (2014a) because it provides a clearly defined set of heuristics that could be applied to a rigorous content analysis of supervisors’ feedback, which would answer the question: how does supervisors’ written feedback in the setting of ‘real-time’ clinical practice construct the CanMEDS roles? We were aware that we might miss some aspects of discourses that were only discussed orally during feedback conversations; focusing on written feedback, however, allowed us to incorporate many different feedback comments from many supervisors to many residents in a detailed textual analysis, which would not be possible when analysing feedback conversations.

## Methods

### Ethics approval

At the time this study was set up, educational research was exempt from institutional board review in the Netherlands. The research was designed to meet the Helsinki Declaration guidelines (Eva 2009; ten Cate 2009; WMA 2008). Naturalistic data—feedback forms collected in usual practice—were used. Both residents and supervisors consented to participate. Residents submitted copies of their feedback forms, which were then anonymised by the researcher.

### Procedure and Participants

Data were collected from seven teaching hospitals affiliated to the internal medicine residency programme of the University Medical Centre, Groningen, the Netherlands. Routine feedback forms from all 120 first, second and third year residents, and all their 126 supervisors were included. Programme directors informed the supervisors and residents about the study and the researchers held a short introductory meeting at the beginning of the data collection period to explain the feedback method used and the procedures of the study. During the course of 1 year, residents handed in anonymised copies of all their feedback forms to departmental secretaries, who forwarded them in sealed envelopes to the primary researcher (NR).

The CanMEDS framework has been used in Dutch Internal Medicine postgraduate training programmes since 2001. Stakeholders, including programme directors from Internal Medicine departments, slightly adjusted the original CanMEDS framework to make it suitable for the Dutch internal medicine context. The role *Professional* was changed into *Reflective Professional* (Gans 2009). The underlying competencies of the roles remained largely the same. All internal medicine departments in the Netherlands use the adjusted framework. Supervisors were offered ‘Teach-the-teacher’ training on how to give feedback in a competency-based setting.

Workplace-based assessments in this postgraduate programme were guided by the principle that versatile roles should be evaluated in different authentic settings. Experienced programme directors assigned different CanMEDS roles to authentic settings that are suitable for direct observation and feedback. Feedback was to be given after direct observation of a resident’s performance during patient encounters, on-calls, clinical handovers, presentations of a critical appraisal of a topic (CAT), or oral presentations. These settings influence the topics that are discussed in the feedback conversations, and therefore affect how the roles are constructed in our data. A broad variety of those feedback settings were included in this study, in an attempt to capture the broad discourse of CanMEDS roles as it occurs in the clinical practice. Table 1 describes how the different roles were evaluated in the various settings of evaluation. Feedback was given orally shortly after residents’ performance had been directly observed. Supervisors were instructed to write down both what went well and what could be improved per CanMEDS role, on feedback forms specific to the different settings. That written feedback was used for this study. Both supervisors and residents could initiate feedback sessions. The forms were held in residents’ portfolios.

**Table 1:** Description of authentic settings in which the CanMEDS roles were evaluated

Medical expert	*Patient encounter:* the resident diagnoses or treats a single patient
Communicator	*Patient encounter:* the resident has one on one contact with a patient (sometimes including their family) in order to diagnose, discuss treatment options or share results of diagnostic tests *On*-*call*: the resident has been on-call over a weekend during which he/she was responsible for all patients admitted to the medical ward and conducted emergency consultations. *Communicator* here focuses not on single patient encounters, but on communication processes with staff, patients and others over the weekend *Morning report*: in this setting, the resident’s task is to safely hand over patients from the night shift. This regards communicating with staff about patients *Critical appraisal of a topic (CAT)*: the resident prepares a presentation to other residents and supervisors about a patient problem that emerged from practice. Results of a literature review and suggestions for treatment plans are presented *Oral presentation*: a relevant published study is presented to fellow residents and supervisors, to stimulate discussion about how it contributes to other studies on the same topic. This setting is different from a *CAT* in that it is not related to a single specific patient problem
Collaborator	*On*-*call*: supervisors evaluate how well the resident has collaborated with nurses, fellow residents, senior staff, consultants and patients during a weekend on-call *Morning Report:* the resident collaborates by providing information to the right people and dividing out treatment tasks
Scholar	During *Critical Appraisal of a Topic* and *Oral Presentations,* the resident is evaluated on scholarly skills such as formulating scientific questions, performing literature searches and critically appraising the literature. The contribution to evidence-based practice is to identify how scientific findings can contribute to the practice of patient care
Manager	*On*-*call:* management is evaluated during a weekend shift in which a resident has to divide their time over all the patients, handle emergencies, prioritise patient problems and divide tasks between members of the team
Reflective professional	*Morning report:* the resident has to reflect on what they did with their patients during the night shift, why they did it and what the next steps should be when handing them over to the next shift. Leading the care-transition in a professional, effective way is evaluated here *CAT/Oral presentation*: the resident has to reflect on scientific information, its value and its implications for practice, and present this in a professional manner
Health advocate	Health Advocate was not part of the regular feedback system in the context of our study, and is therefore not reflected in our dataset

### Research team

The research team was assembled to represent a range of disciplines, which could inform the data interpretation. NR is a Ph.D. candidate in medical education that has a master’s degree in educational sciences. TD is an experienced internist and expert in qualitative research. RG is a medical doctor and programme director in Internal Medicine who helped implement the CanMEDS framework in the Netherlands. JB is a medical doctor and dean of education in a University Medical Centre. JC is a psychologist and professor in medical education. DJ is a professor in medical education whose background is in veterinary medicine. NR, RG, JB, DJ and JC are all native Dutch speakers, whereas TD is a native English speaker who is able to read Dutch. TD used his relative unfamiliarity with the Dutch language to look critically at the written comments, which helped the research team maintain a questioning attitude towards how language was used.

### Analysis

*Health advocate* was not addressed in the feedback system in this setting, therefore the researchers did not have data on this role. Since physicians have always been expected to be medical experts and scholars, formalising the non-technical roles of *Communicator*, *Collaborator*, *Manager*, and *ReflectiveProfessional* is a novel feature of CanMEDS. The research team therefore chose to concentrate its analysis on those four roles.

NR first made herself thoroughly familiar with the data by reading and re-reading all written comments on all forms. Since the dataset consisted of 3150 written feedback comments on 591 feedback forms and the sheer quantity of data can compromise the quality of critical discourse analysis, NR purposefully selected a subset of data that was likely to be most informative. First she excluded all forms with comments that were not amenable for textual analysis because they were too short, fragmentary, or general; e.g. ‘well done’. After that, she purposively chose the 100 forms (20 from each feedback setting) with 297 written comments, which best illustrated the variation in the texts as the dataset for analysis.

NR and TD led the application of a set of fine-grained critical discourse analysis “tools” (Gee 2014b) to draw attention to significant statements, key words, and metaphors. Two main tools that were used are explained below.

1. **‘**Doing and not just saying**’** This tool emphasises the notion that any piece of textual communication consists of more than what is literally said. It examines the social effects that each piece of text might be expected to have. For example, a supervisor who commented that a resident had used over-familiar language when speaking to patients was creating greater professional distance between physician and patient.
2. *Significance* this tool can be applied to analyse how language increases the significance of certain things and reduces the significance of others. This was exemplified in the dataset by, for example, underlining words, using punctuation such as exclamation marks or brackets, and sentence structure. The following suggestion for improvement illustrates how significance was reduced: ‘No suggestions. But I would like to point out a pitfall: be aware that you could come across business-like to patients (not necessarily a disadvantage)’ The feedback comment begins by stating there are no suggestions for improvement, continues with a formative statement, and ends by saying that the observed behaviour is not necessarily a disadvantage. The net effect of this comment is to sanction ‘coming across business-like’ within the discourse of medical communication. This example also shows how our discourse analysis was ‘critical’, because it shows how coming across to patients as ‘business-like’ is part of the relationship the supervisor builds with the supervisee within the institution of medicine.

First, TD and NR individually open-coded the data concerning *Communicator* and *Collaborator* of a random subset of 25 forms—five from each setting—by applying Gee’s tools. They read the texts closely and open-coded them with words or short sentences that captured meaning(s) they found in the text. They constantly compared (Glaser and Strauss 2012) their findings and agreed that supervisors were inconsistent in whether they attributed similar observations to the roles of *Communicator* or *Collaborator,* which they seemed to use interchangeably. Whilst supervisors’ blurring of the two roles was contrary to the intention of CanMEDS, it was so difficult to distinguish them that the researchers decided to put all comments on these roles together for coding, gradually added other roles, and finally arrived at an agreed coding framework for all roles.

Second, NR applied the agreed coding framework to the remaining data. She then re-sorted the data per role to explore different discourses in the data. She discussed any ambiguities and her findings as a whole with DJ and TD.

Third, all researchers discussed the coded dataset, arriving at preliminary conclusions about how the roles were constructed. NR then re-read all 591 forms to check that important findings were not missing from the purposively selected subset of forms.

Finally, since the researchers had become interested in how the discourses seemed to construct only a peripheral role for patients in the preceding steps, they decided to conduct a fourth and final step. They identified all comments referring to patients, first in the purposive sample and then in the complete dataset. From the complete dataset, feedback provided during 32 Patient Encounters, 12 On-Calls and 11 Morning reports was selected and analysed additionally. NR coded these comments, the researchers collectively agreed how to interpret their meaning.

### Presentation of results

The findings are organised by CanMEDS role, drawing together the discourse of each role within the whole dataset. To illustrate our findings, we include quotes, each of which is the full text of an item of feedback for a specific role in a specific setting. Translations of the comments from Dutch to English have been agreed between native speakers of the two languages.

## Results

### Communicator

#### Communicating with patients

The discourse for communicating with patients was constructed with a strong emphasis on time-efficiency; for example, finishing a consultation on time. Compassionate aspects of communication, such as showing empathy and comforting, were often coupled with the need for speed: “Listens well in order to ask smart questions based on the limited information the patient provides. Clearly repeats patients’ conclusions to verify. Comforts a—slightly distrustful- patient in a very good/high pace.” and: “Discovered underlying problem well (dead child). Ensured good speed (truncates a clarifying answer in a friendly way)”.

The discourse included how residents came across: “Be aware of your use of language (too familiar)”. It also included how they created room to involve patients in conversations: “Held a bad news conversation in a calm, empathic, manner with family + patient. Created space for questions and expression of emotions”.

#### Communicating with staff

The discourse of communicating with staff also strongly emphasised time-efficiency, being to-the-point, concise and clear, and giving just enough detail without wasting time: “Your narrative explanation of the essence of the handover sheet was concise and to the point. Despite high speed, good contact with audience”. Other features of the discourse were, on the one hand, being assertive and exercising leadership, and on the other cooperating and engaging others. *Being assertive* meant being pro-active, delegating tasks, and telling others what to do. Exercising leadership meant showing responsibility, being in control, and approaching the right people. In the following text, the supervisor equated the metaphor of ‘being king’ with leadership “Be concrete about the policy. Hand over to the ward. Looking ahead would make you king”. Cooperation was constructed in terms of being approachable, conferring, and liaising with others: “Critical about colleagues’ and own performance; but now it is time to propose treatment plans yourself”. Engaging included having good contact with others and inviting others to contribute to discussions: “Focus your presentation (especially during morning reports) a bit more on what you consider to be essential issues. Invite others to be more engaged in the discussion and provide you with feedback”.

### Collaborator

There were two different discourses of *Collaborator:* a consensual one and one that constructed an imbalance of power in favour of physicians. The consensual *Collaborator* was a cooperative individual, ready to work with others. Exercising leadership by taking the initiative and being assertive were part of this discourse: “Pleasant collaboration, gives a sense of a joint undertaking. Great sense of responsibility. Clear communication”. The alternative discourse placed residents in positions of power, as people who were in charge of other people and had to defend their position against others: “If people do not do what you want, I think you find it difficult to take a “firm” stance”. This discourse constructs a firm, directive type of leadership: “Work on limiting your responsibility and, if necessary, “resistance”. Articulate assignments more clearly, be more assertive and directive”.

### Manager

The discourse constructed three facets of a *Manager:* ‘managing oneself’, ‘managing others’ and ‘managing means’. ‘Managing oneself’ meant having fore-, back-, and over-sight, being meticulous, and knowing and respecting one’s own boundaries: “Try to do important things first (diagnostics). Make lists, try to schedule tasks. Complete one thing so you can start the next one. Try to be a bit faster.” ‘Managing others’ meant managing staff, knowing when to consult or check others, and being directive and delegating tasks: “Extra attention to triage/prioritizing. Don’t do everything yourself but allocate/delegate tasks in a directive way!” ‘Managing means’ included deliberately choosing diagnostic tests according to how they could contribute to a treatment policy whilst keeping unnecessary tests to a minimum: “Considers advantages and disadvantages, usefulness of diagnostic tests”. Being goal-directed and taking the initiative in making policy and treatment plans was a consistent feature of the discourse of *Manager.* The *Manager* role also constructed physicians as people who work fast and prioritize tasks so everything is completed in time.

### Reflective professional

The discourse of *Reflective Professional* constructed residents as trainees, members of healthcare teams, and all-round professionals. Residents ‘as trainees’ were to ask for feedback, ask for help, seek to improve themselves, and know their limitations: “Maintains good oversight of a broad differential diagnosis. Presents himself as inquisitive and keen to learn (open). Very good discussion of 2 (more) complex cases”. Residents ‘as team members’ were professionals who were open and approachable, conferred with others, made decisions in dialogue, but also stood up for themselves and took the lead in discussions: “Remain critical about patients that are handed over from other colleagues. Is everything correct? Relevant and logical?” Residents ‘as all-round professionals*’* managed time, were critical, had oversight and went deep into problems to make a working diagnosis or treatment plan: “Fine attitude. Dares to go back to the beginning when hits a dead end”.

### The patient’s role

Patients were relatively rarely mentioned, even when the analysis was extended to the roles of medical expert and scholar. Although patients were not privy to some settings, such as CAT and Oral presentation, settings such as Patient Encounters and On-Call directly involved patient interactions. The absence of patients in these data, whilst a finding in its own right, limits the depth to which the analysis could be taken. Patients were part of the discourses of *Communicator, Collaborator,* and *Medical Expert,* but not even mentioned in relation to *Manager, Reflective Professional,* and *Scholar.* The relationship between residents and patients was explicitly distant: “No suggestions. But I would like to point out a pitfall: be aware that you could seem business-like to patients (not necessarily a disadvantage)” and: “Is approachable to other professionals. Clear to patient.“The discourse of *Communicator* included communicating with patients in friendly ways, and making patients *feel* comfortable and understood, rather than *being* comfortable or understood: “Reflect on patient’s feelings so they will feel better understood”. Patients tended to be positioned as subjects of communication or collaboration rather than equal participants. The word ‘listen’ was strikingly absent from the discourse—it was used just once in 3150 items of text.

## Discussion

### Principal findings and meaning

This study has shown that the discourse of physicians, in the authentic workplace settings we studied, constructs the CanMEDS roles in very different ways from what the originators of CanMEDS might have intended. There were three striking differences. First, there was a discourse of collaboration according to which residents were directive towards others, exercising a firm kind of leadership. Although directive types of leadership can be effective in critical or extremely high-workload settings, empowering leadership styles function better in creating a positive climate and increasing cohesion within functioning teams (Kuenzle et al. 2010). Second, the discourses of all CanMEDS roles were dominated by efficiency; being fast was considered essential. Even aspects of patient communication, such as showing empathy and asking questions, were counterpoised with comments on speed. This finding should be taken into account in on-going conversations about healthcare systems, which have to become more efficient whilst, at the same time, paying more attention to non-technical skills and patient-centred behaviour. Third, physicians’ contribution did not go beyond behaving kindly towards patients; patients were positioned as objects in the periphery rather than participants at the centre of care. This is an important finding since patient-centred approaches and mutual, reciprocal, relationships between patients and physicians are associated with a higher quality of care (Mead and Bower 2000; Bleakley 2014).

There were other discourses that were in line with what was intended in the original framework. The discourse of Manager emphasised ordering diagnostic tests thoughtfully and not being wasteful whilst gathering enough information to make diagnoses and keep patients safe. CanMEDS describes its roles as intertwined with one another, which is in line with how supervisors constructed them as rather blurred. Although some roles (Reflective Professional, Manager) were constructed in a more limited fashion in our data than in the original CanMEDS framework, this was often compensated for by feedback on other roles. It appears that there is some unifying discourse of ‘a good physician’ that can be viewed through different competency spectacles to highlight certain aspects of practice or performance. This aligns well with common critique that in the CanMEDS framework the complex profession is rather artificially divided into seven roles, whereas the reality is that roles overlap and complement each other (Whitehead et al. 2013).

### Strengths and limitations

A distinctive feature of this study was the detailed textual analysis of relatively fragmented data. Pressure of time obliged physicians giving feedback to express what they saw as key points in limited words, and therefore the fragmentary data indicate what was considered most important. Another strength is that these data are the original feedback that the residents received. Although other methods, such as interviews or focus groups, might result in a more complete articulation of the discourse, these methods would also allow for more socially desirable responses whereas our naturalistic data reflect the actual process-in-action. We must acknowledge, however, that although we received feedback forms from all 120 residents, we are not sure whether they submitted all their feedback forms for inclusion in our analysis, so some unseen bias might have operated.

The varied backgrounds and roles taken on by members of the research team in analysing the data increased the rigour in this study. Qualitative analysis is an inherently subjective process, and we cannot guarantee that a different team would have arrived at the same conclusions. However, we worked together as a team, capitalising on how our different backgrounds gave different perspectives on the data.

Our conclusion that patients were marginalised in the discourse was undoubtedly influenced by the data having captured conversations between physicians. Whilst we can draw no conclusions about the conversations that might have gone on between a teacher, a resident, and a patient, we can draw the conclusion that the discourse of the CanMEDS roles in this context does not give patients a central place.

### Implications for practice and future research

This example of how outcomes-based education operates in practice highlights that defining learning outcomes and using them to structure a curriculum does not automatically result in the intended changes in practice. Although the qualitative methods we used in this study do not aim to produce generalizable results, readers who recognise similarities between their workplace settings and ours may find them transferable to their own competency-based training programmes (Kuper et al. 2008). In that case, greater engagement of supervisors in the development of competency frameworks and training to help them better understand the roles, would be appropriate steps to take. Supervisors’ feedback could benefit from making the original goals of CanMEDS more explicit. However, we cannot say how effective these actions would be without conducting further research.

Another avenue for future research would be to study how the role of *Health Advocate* is constructed in practice. The feedback system used in this study did not generate feedback on this role. Analysing the discourse of this role in practice and especially the position of patients within it could complement this study.

## Conclusions

It is striking that supervisors’ discourses constructed a tension between being patient-centred and being time-efficient. Since we do not know whether that tension is inevitable, it is a matter for further research. It is questionable, however, whether such a high-pressure environment is a fitting place to engender patient-centred attitudes. It seems challenging for residents to combine the roles of a well-functioning physician and a trainee in a setting that focuses so strongly on being fast and to the point. Asking a supervisor to spend valuable time to explain something or observe and give feedback in such a time-pressured setting could be difficult for residents. Spending as little time as possible with a patient while trying to manage to listen, comfort and deliver high quality care is certainly a challenge.
